# Impact of a trace mineral injection at weaning on growth, behavior, and inflammatory, antioxidant, and immune responses of beef calves

**DOI:** 10.1093/tas/txae177

**Published:** 2024-12-26

**Authors:** Marcelo Vedovatto, Matheus F L Ferreira, Ashley K Edwards, Jeffrey A Gurie, Hiam Marcon, Juliana Ranches, Barbara R Reis, Douglas G Vieira, Eduardo A Lima, Mariana Santos, Gumercindo L Franco

**Affiliations:** Dean Lee Research and Extension Center, Louisiana State University, Alexandria, LA 71302, USA; Hill Farm Research Station, Louisiana State University, Homer, LA 71040, USA; Dean Lee Research and Extension Center, Louisiana State University, Alexandria, LA 71302, USA; Hill Farm Research Station, Louisiana State University, Homer, LA 71040, USA; Dean Lee Research and Extension Center, Louisiana State University, Alexandria, LA 71302, USA; Dean Lee Research and Extension Center, Louisiana State University, Alexandria, LA 71302, USA; Eastern Oregon Agricultural Research Center, Oregon State University, Burns, OR 97720, USA; CREC-White Sand Beef Unit, Mississippi State University, Poplarville, MS 39470, USA; Faculdade de Medicina Veterinária e Zootecnia, Universidade Federal de Mato Grosso do Sul, Campo Grande, MS 79074-460, Brazil; Faculdade de Medicina Veterinária e Zootecnia, Universidade Federal de Mato Grosso do Sul, Campo Grande, MS 79074-460, Brazil; Unidade Universitaria de Aquidauna, Universidade Estadual do Mato Grosso do Sul, Aquidauana, MS 79200-000, Brazil; Faculdade de Medicina Veterinária e Zootecnia, Universidade Federal de Mato Grosso do Sul, Campo Grande, MS 79074-460, Brazil

**Keywords:** cortisol, haptoglobin, Multimin, stress, superoxide dismutase, vaccine

## Abstract

Two experiments evaluated the effects of an injectable trace mineral (ITM) solution at weaning on trace mineral (TM) status, inflammatory and antioxidant responses, grazing behavior, response to vaccination, and growth of beef calves. Experiment 1 used 86 Nellore calves (40 females and 46 males; body weight [BW] = 198 ± 30.8 kg; 8 ± 1 mo of age) weaned (day 0) and assigned into one of two treatments: saline (0.9% NaCl) or ITM (60 mg of Zn/mL, 15 mg of Cu/mL, 5 mg of Se/mL, and 10 mg of Mn/mL). Saline and ITM were administered subcutaneously at a dose of 1 mL/45 kg of BW. On day 0, calves were vaccinated against infectious bovine rhinotracheitis (IBR), parainfluenza-3 (PI_3_), bovine viral diarrhea virus types 1 and 2 (BVDV-1 and 2) and bovine respiratory syncytial virus (BRSV). Blood samples and BW were collected on days 0, 3, 8, 15, 51, and 100, and grazing behavior was evaluated on days 1, 2, 4, 5, 6, 7, and 9. The ITM did not affect (*P* ≥ 0.56) serum mineral concentrations of Zn and Cu, but decreased (*P* ≤ 0.02) plasma concentrations of cortisol on days 3 and 8 and haptoglobin on day 3. The ITM increased (*P* < 0.01) plasma concentration of superoxide dismutase on days 8, 15, and 51 and tended to decrease (*P* = 0.08) plasma concentration of glutathione peroxidase on day 3. Furthermore, there were no effects of treatment (*P* ≥ 0.14) on most of the behavior variables evaluated, ITM reduced (*P* = 0.01) the seeking time on days 0 and 4. Furthermore, ITM tended to increase (*P* = 0.10) the serum titer concentration against IBR on days 15 and 51 but did not affect (*P* ≥ 0.12) titer concentration against PI_3_, BVDV-1, and 2, and growth. Experiment 2 used 50 Brangus male calves (BW = 264 ± 34.1 kg; 8 ± 1 mo of age) weaned on day 0, vaccinated against respiratory diseases, stratified by BW, and randomly assigned to saline or ITM as described in experiment 1. Liver samples were collected on days 0, 14, and 197, blood samples on days 0, 14, and BW on days 0, 14, 44, 78, 122, 162, and 197. The ITM increased (*P* ≤ 0.03) the liver concentration of Cu and Se on day 14 but did not affect (*P* ≥ 0.17) the liver concentration of Zn and Mn. In addition, ITM increased (*P* = 0.05) the serum titer concentration against BVDV-2 but did not affect (*P* ≥ 0.20) the titer concentrations against IBR, PI_3_, BVDV-1, and BRSV, and did not affect (*P* ≥ 0.29) the growth. In conclusion, ITM application at weaning improved Cu and Se status, enhanced antioxidant and immune responses, and reduced stress and inflammation in calves, though it did not affect growth.

## Teaser

Applying an injectable trace mineral (ITM) solution at weaning improved the Cu and Se status of calves, reduced stress and inflammation, and improved the antioxidant response; these effects led to a better response to the vaccination but did not affect the growth. Improving the immune system by the application of ITMs could be important for operations that face health issues in their animals after weaning.

## INTRODUCTION

Weaning is a crucial management practice in beef cattle operations that exposes calves to various stressors. These stressors can result in adrenocortical and acute-phase responses leading to negative effects on the performance, health, and immunity of cattle (Vieira et al., 2023). During this critical period, cattle require greater amounts of specific trace minerals (TM) to mount an effective immune response and maintain adequate growth (NASEM, 2016; Arthington and Ranches, 2021). Specifically, TM such as Zn, Cu, Se, and Mn play an essential role in growth (Spears and Kegley, 2002), proliferation of immune system cells (Maggini et al., 2007), as well as serving as components of antioxidant enzymes (Rotruck et al., 1973; Sordilo and Aitken, 2009).

For grazing cattle, free-choice mineral supplementation is usually the primary and most common source of TM (Arthington et al., 2014), as forage often does not provide adequate amounts; nonetheless, the efficacy of free-choice minerals supplementation is impacted by high variation in consumption. ITM is a technology that assures delivery of a known quantity of essential TM, increasing short-term TM status (Arthington and Havenga, 2012; Pogge et al., 2012). An additional supply of minerals with ITM during challenging production periods, such as weaning, has been shown to improve humoral and cell-mediated responses after a viral vaccination protocol (Arthington et al., 2014), antioxidants enzyme activities (Vedovatto et al., 2020), and reduce morbidity rates (Richeson and Kegley, 2011). However, literature data on growth performance and stress-related responses to the supplementation of ITM are still inconsistent. Some studies have reported greater growth performance in calves supplemented with ITM at weaning (Richeson and Kegley, 2011; Rauch et al., 2019), while others have found no differences (Vedovatto et al., 2020; Hernandez et al., 2024), or even reduced growth performance (Arthington et al., 2014; Zornitta et al., 2022) compared to control groups. Additionally, there are no conclusive results regarding stress and inflammatory response after ITM application on calves after weaning (Arthington et al., 2014; Genther-Schroeder and Hansen, 2015; Caramalac et al., 2021). The variability of ITM responses in different studies could be attributed to many factors, such as the magnitude of stress, breed, and nutritional and TM status.

Furthermore, while the majority of data on ITM has been developed using *Bos taurus* cattle newly received on a feedlot, data on *Bos indicus* (e.g., Nellore) or *Bos Indicus*-influenced breeds (e.g., Brangus) under a pasture-based system is scarce. Given that breed can also influence TM metabolism (Mullis et al., 2003; Pogge et al., 2012; Ranches et al., 2021), more studies are warranted in this regard since it may influence the ITM effect on performance and physiological responses.

In an experiment conducted by Rauch et al. (2019), the ITM application at weaning affected the behavior of the calves, increasing the time they spent walking after weaning. The authors could not elucidate the underlying cause of this outcome, so we also decided to investigate the effects of ITM on behavior and stress further. Therefore, we hypothesized that ITM application in calves would increase TM status, improve antioxidant and immune responses, grazing behavior, and growth, and reduce stress after weaning. Thus, two experiments were conducted to evaluate the effects of ITM application at weaning on TM status, inflammatory and antioxidant responses, behavior, response to vaccination, and growth of beef calves.

## MATERIAL AND METHODS

Experiment 1 was conducted at the farm school of the Universidade Federal de Mato Grosso do Sul (UFMS), Terenos, MS, Brazil (20°26’50.8”S 54°50’21.5”W) and approved by the UFMS Animal Care and Use Committee (# 754/2016). Experiment 2 was conducted at Dean Lee Research and Extension Center (Alexandria, LA, USA; 31°10’15.64”N 92°24’5.30”W) and Iberia Research Station (Jeanerette, LA, USA; 29°57’32.52”N 91°42’40.76”W) from the Louisiana State University Agricultural Center (LSU AgCenter) and approved by the LSU AgCenter Animal Care and Use Committee (# A2023‐10).

### Experimental Design and Calf Management

#### Experiment 1.

In experiment 1, 86 Nellore calves [40 females and 46 males; body weight (BW) = 198 ± 30.8 kg; 8 ± 1 mo of age] were abruptly weaned on day 0, stratified by BW and sex, and randomly assigned into 1 of 2 treatments: Saline injection (0.9% NaCl) or ITM. The ITM solution composition consisted of 60 mg of Zn/mL, 15 mg of Cu/mL, 5 mg of Se/mL, and 10 mg of Mn/mL (Multimin 90, Axiota Animal Health, Fort Collins, CO, USA). Saline and ITM were administered subcutaneously at a dose of 1 mL/45 kg of BW on the right side of the neck of each calf. On day 0, before treatment application, calves were vaccinated against infectious bovine rhinotracheitis (IBR), parainfluenza-3 (PI_3_) virus, bovine viral diarrhea virus type 1 (BVDV-1) and 2 (BVDV-1), bovine respiratory syncytial virus (BRSV), and *Mannheimia haemolytica* (2 mL s.c.; Bovi Shield Gold One Shot, Zoetis, São Paulo, SP, Brazil) and no booster was applied.

From days 0 to 15, calves were maintained in a single 2-ha pasture of Marandu-grass (*Brachiaria brizantha* [*Syn. Urochloa brizantha*] cv. Marandu) complemented with hay (*Brachiaria humidícola* [*Syn. Urochloa humidícola*] cv. Llanero [Dictyoneura]). On day 15, calves were moved to a larger paddock (11 ha) of Marandu-grass, where they were kept until the end of the study (day 100; Table 1). Calves had free-choice access to water and energy-protein supplement (guaranteed levels of 30% of crude protein [CP], 24.6% of non-nitrogen protein, 7.5% of Na, 4.52% to 5.28% of Ca, 0.73% of P, 0.75% of S, 200 mg/kg of Cu, 133 mg/kg of Mn, 600 mg/kg of Zn, 12 mg/kg of Co, 15 mg/kg of I, 3.6 mg/kg of Se, and 160 mg/kg of monensin; target intake of 1 g/kg of BW; Recritech 100, Techagro Nutrição Animal, Campo Grande, MS, Brazil).

**Table 1.** Chemical composition of forages and hay of experiments 1 and 2^1^

Items	Experiment 1 Forage				Experiment 1 Hay	Experiment 2 Forage							Experiment 2 Hay	Requirements (NASEM, 2016)^2^
	Day 0	Day 15	Day 51	Day 100		Day 0	Day 15	Day 44	Day 78	Day 122	Day 162	Day 197		
Crude protein, %	8.50	7.85	9.10	8.30	10.2	7.60	8.20	8.80	8.60	10.0	20.5	10.3	10.0	
Neutral detergent fiber, %	62.1	67.4	64.5	66.3	75.9	62.3	64.4	64.5	65.5	70.0	41.6	53.6	70.0	
Acid detergent fiber, %	33.8	39.5	35.4	29.7	40.3	32.0	37.4	35.5	39.0	38.3	27.7	34.0	38.3	
Calcium, %	0.42	0.42	0.50	1.00	0.31	0.51	0.43	0.54	0.40	0.50	0.58	0.46	0.50	
Phosphorus, %	0.24	0.11	0.10	0.50	0.20	0.30	0.21	0.24	0.21	0.24	0.48	0.38	0.24	
Potassium, %	1.50	1.37	1.14	1.41	1.84	1.69	1.49	1.75	0.87	1.59	4.08	2.95	1.59	0.60
Magnesium, %	0.27	0.33	0.34	0.27	0.15	0.21	0.38	0.21	0.16	0.24	0.23	0.20	0.24	0.10
Zinc, mg/kg	10.8	18.7	23.6	16.6	26.7	43.0	48.0	51.0	73.0	44.0	29.0	32.0	44.0	30.0
Copper, mg/kg	3.77	5.23	11.1	4.01	4.86	6.00	8.00	8.00	10.0	9.00	9.00	6.00	9.00	10.0
Selenium, mg/kg	0.12	0.11	0.13	0.10	0.07	2.30	0.44	2.17	1.10	0.71	0.07	0.21	0.72	0.10
Manganese, mg/kg	142	166	219	153	53.9	48.0	191	79.0	188	64.0	160	149	64.0	20.0
Iron, mg/kg	49.6	55.1	47.6	51.5	62.4	241	364	175	504	400	211	580	400	50.0
Total digestible nutrients^3^, %	59.6	53.7	55.0	59.3	55.25	60.0	58.9	59.1	59.0	54.0	65.0	62.0	54.0	
NEm^4^, Mcal/kg	1.30	1.10	1.14	1.29	1.15	1.31	1.28	1.28	1.19	1.11	1.47	1.30	1.11	
NEg^5^, Mcal/kg	0.72	0.54	0.58	0.71	0.59	0.74	0.71	0.71	0.63	0.55	0.86	0.88	0.55	

^1^Experiment 1 was conducted at the farm school of the UFMS, Terenos, MS, Brazil, and used 86 weaned Nellore calves (40 females and 46 males). Experiment 2 was conducted at Dean Lee Research and Extension Center (Alexandria, LA, USA) and Iberia Research Station (Jeanerette, LA, USA) from Louisiana State University AgCenter and used 50 weaned male Brangus calves.

^2^Requirements for growing calves were established by NASEM (2016).

^3^Calculated as described by Weiss et al. (1992).

^4^NEm and NEg were calculated using the equations proposed by the NASEM (2016).

#### Experiment 2.

Experiment 2 started at Dean Lee Research and Extension Center using 50 Brangus male calves (BW = 264 ± 34.1 kg; 8 ± 1 mo of age] weaned on day 0, stratified by BW and randomly assigned into one of two treatments: Saline and ITM as described in experiment 1. On day 0, before treatment application, calves were revaccinated (first vaccination was conducted 30 d before weaning) against respiratory diseases (Bovi Shield Gold One Shot, Zoetis) as described in experiment 1. Differently from experiment 1, these calves were fence-line weaned for 14 d (days 0 to 14) in a single 5.4-ha pasture of Bermuda grass (*Cynodon dactylon*), supplemented with Bermuda grass hay (Table 1) and free-choice mineral supplement (guaranteed levels of 13.5% to 16.2% of Ca, 7.50% of P, 17.1% to 20.5% of NaCl, 1% of Mg, 1% of K, 4,800 mg/kg of Mn, 18 mg/kg of Co, 2,500 mg/kg of Cu, 60 mg/kg of I, 27 mg/kg of Se, 7,500/kg of Zn, 330,000 IU/kg of vitamin A, 33,000 IU/kg of vitamin D3, and 330 IU/kg of vitamin E; target intake of 113 g/head per day; Purina Wind and Rain Storm TX All Season 7.5 complete [Land O’Lakes, Inc., Arden Hills, MN, USA]).

On day 15, calves were trailer transported for 167 km to Iberia Research Station, where they remained in a single group grazing Bermuda grass from days 15 to 122 and then Ryegrass (*Lolium multiflorum*) forage until day 196. Animals were rotated weekly in four 5-ha pastures. Bermuda grass hay and a free-choice mineral formula (Purina Wind and Rain Storm TX All Season 7.5 complete; Land O’Lakes, Inc.) were provided throughout the experiment.

### Data and Sample Collection

#### Experiment 1.

Full BW of calves were collected individually at 0800 h on days 0, 8, 15, 51, and 100. Shrunk BW was not obtained to avoid shrink-induced stress effects on blood parameters evaluated in the study (Marques et al., 2012). Furthermore, on day 0, calves were individually identified on both sides of the body, with large numbers, using hair dye. An observation tower (elevated 5 m from the ground) was used by the evaluators to facilitate the visualization of the animals. The diurnal behavior of the animals was evaluated from 0630h to 1800h; with a 10-minute interval between each scan, on days 1, 2, 4, 5, 6, 7, and 9. The variables evaluated were adapted from Enríquez et al. (2010): grazing, eating concentrate, drinking water, walking, lying, lying ruminating, standing, standing ruminating, playing, vocalizing, and seeking. Further, the variables total lying (lying + lying ruminating), total standing (standing + standing ruminating), and total ruminating (lying ruminating + standing ruminating) were calculated. A list describing the definition of each behavior evaluated is presented in Table 2.

**Table 2.** List of behaviors observed and their respective description

Items^1^	Definition
Grazing	Picking or consuming pasture, with the head close to the ground, still, or moving slowly
Eating concentrate	Mouth below the feedline in the trough ingesting concentrate
Drinking water	Mouth below the waterline in the trough ingesting water
Walking	All four legs were moving with the head raised or not (still)
Lying	Lying down in any resting position
Lying ruminating^2^	Lying down in any resting position and ruminating
Standing	Not walking
Standing ruminating^2^	Not walking and ruminating
Playing	Jumping, running, with no sign of stress
Vocalizing	Making sounds and heard by the observer
Seeking	Walking beside the fence, with head up, looking for the dam

^1^Adapted from Enríquez et al. (2010).

^2^Ruminating was defined as chewing regurgitated boluses of feed.

Blood samples were collected from a jugular vein (14 calves/treatment; 7 females and 7 males/treatment) on days 0, 3, 8, 15, 51, and 100 into two blood collection tubes (10 mL; Vacutainer, Becton Dickinson, Franklin Lakes, NJ, USA) with and without sodium heparin, for collection of plasma and serum, respectively. After collection, blood samples were immediately stored on ice and then centrifuged at 1,200 × g for 30 min for plasma and serum harvest. Serum and plasma samples were stored at –20 °C for further analysis. Serum samples were analyzed for cortisol, and plasma samples for haptoglobin, ceruloplasmin, superoxide dismutase (SOD), and glutathione peroxidase (GSH-px) concentrations on days 0, 3, 8, 15, 51, and 100. Serum samples were also analyzed for Cu and Zn concentrations, and antibody titters against IBR, PI_3_, BVDV- 1, and BVDV-2 on days 0, 15, and 51.

Hand samples of forage and hay were collected on days 0, 15, 51, and 100. Afterward, samples were dried in a ventilated oven at 60 °C for 5 d and ground at 1 mm for later chemical and mineral composition analysis. A composite sample of hay was made before analysis.

#### Experiment 2.

Liver samples were collected on days 0, 14, and 197 as described by Hernandez et al. (2024). Briefly, liver samples were collected between the 10th and 11th intercostal space using a Tru-Cut biopsy needle (CareFusion, 14-gauge × 15 cm; Becton Dickinson, Vernon Hills, IL, USA). Four core tissue samples were collected from each animal. Following collection, samples were frozen at −20 °C and sent to an analytical laboratory for mineral analyses (Michigan State University, Animal Health Diagnostic Laboratory, Lansing, MI, USA).

Blood samples were collected from a jugular vein (12 calves/treatment;) on days 0, 14, and 44 into a blood collection tube (10 mL; Vacutainer, Becton Dickinson, Franklin Lakes, NJ, USA) without sodium heparin for serum collection. Serum samples were processed and stored as described in experiment 1 and analyzed for titer concentration of IBR, PI_3_, BVDV-1 and 2, and BRSV.

Full BW of calves was collected individually at 0800h on days 0, 14, 44, 78, 122, 162, and 197. Hand samples of forage and hay were collected on days 0, 15, 44, 78, 122, 162, and 197 and processed as described in experiment 1.

### Laboratory Analysis

Serum concentrations of Cu and Zn in experiment 1 were analyzed by a colorimetric procedure (Axys Análises, Porto Alegre, RS, Brazil), and liver TM concentrations in experiment 2 were determined via inductively coupled plasma mass spectroscopy (Veterinary Diagnostic Laboratory, Michigan State University, Lansing, MI, USA).

Plasma samples (experiment 1) were analyzed as follows: cortisol concentrations were analyzed by chemiluminescent enzyme immunoassay (Immulite 1000; Siemens Medical Solutions Diagnostics, Los Angeles, CA, USA) and accomplished within a single assay with an intra-assay CV of 8.52%. Haptoglobin concentration was quantified by measuring the differences in peroxidase activity formed by haptoglobin-hemoglobin complexing, as described by Cooke and Arthington (2013). Ceruloplasmin concentration was determined by measuring ceruloplasmin oxidase activity using a colorimetric procedure, as described by Demetriou et al. (1974). The SOD and GSH-px concentrations were determined using commercial kits for ELISA (Cayman Chemical, Ann Arbor, MI, catalog number 703102 and 706002, respectively), The inter- and intra-assay CV were 3.9% and 9.4% for haptoglobin, 2.0% and 4.3% for ceruloplasmin, 4.6% and 6.7% for SOD, and 4.9% and 9.1% for GSH-px, respectively.

Antibody titers against IBR, PI_3_, and BVDV-1 and BVDV-2 viruses were assessed using procedures outlined by Rosenbaum et al. (1970) at the LABVIR laboratory (Santa Maria, RS, Brazil) in experiment 1 and at the Oklahoma Animal Disease Diagnostic Laboratory (Stillwater, OK, USA) in experiment 2 (experiment 2 also evaluates the titer concentrations against BRSV). Individual serum samples were evaluated for the greatest dilution of antibody titers that achieved total cell protection against those viruses.

Forage samples were analyzed according to AOAC (2006): dry matter (method 930.15); CP (method 976.05); ether extract (method 920.39), ash (method 942.05), and minerals (method 985.01). The concentrations of lignin, neutral detergent fiber, and acid (ADF) were analyzed as described by Van Soest et al. (1991). The total digestible nutrients concentrations were calculated as described by Weiss et al. (1992), and net energy for maintenance (NEm) and gain (NEg) by the equations proposed by the NASEM (2016). Commercial laboratories in experiment 1 (Fundação ABC, Castro, PR, Brazil) and experiment 2 (Dairy One Forage Laboratory, Ithaca, NY, USA) analyzed the mineral concentration in forage.

### Statistical Analysis

The calf was considered the experimental unit for all analyses. Normal distribution and homogeneity of variance were analyzed using the UNIVARIATE procedure with the NORMAL option in SAS (version 9.4; SAS Inst. Inc., Cary, NC, USA). Only the serum titer data were not normally distributed (*P* < 0.01; Shapiro-Wilk test) and were log_2_-transformed prior to analysis. Then, all variables were analyzed using the MIXED procedure of SAS with the Satterthwaite approximation to determine the denominator degrees of freedom for the test of fixed effects. All variables were analyzed as repeated measures and tested for fixed effects of treatment, day, and treatment × day. The random effects and subjects were calf (treatment × sex) in experiment 1 and calf (treatment) in experiment 2. The BW and baseline blood and liver data obtained on day 0 were initially included as covariates and tested for significance (*P* ≤ 0.05). The baseline blood and liver data were kept as covariates (*P* ≤ 0.01; i.e., the means reported were adjusted for covariates), but the initial BW was removed (*P* = 0.46) from the model. The first-order autoregressive covariance structure was selected for all variables because it had the lowest values in the Akaike information criterion. Means were separated using PDIFF, and all results were reported as LSMEANS followed by SEM. Significance was defined as *P* ≤ 0.05, and tendency when *P* > 0.05 and ≤ 0.10.

## RESULTS

### Experiment 1.

**Figure 1.**
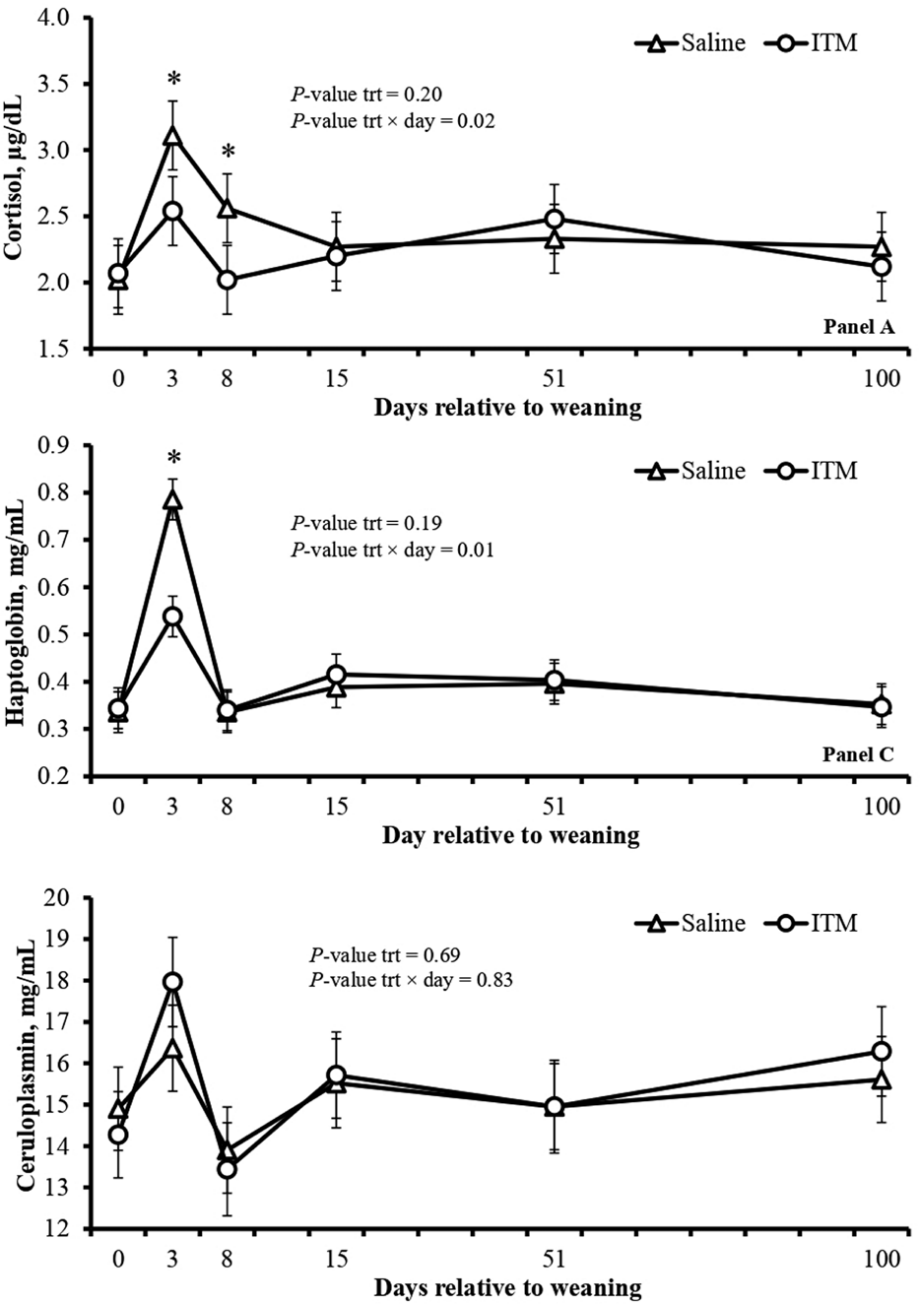
Plasma concentration of cortisol (A), haptoglobin (B), and ceruloplasmin (C) of Nellore calves receiving saline solution (Saline; *n* = 14) or ITM (*n* = 14) at weaning (day 0) in experiment 1. The saline solution consisted of 0.9% NaCl, whereas ITM had 60, 10, 5, and 15 mg/mL of Zn, Mn, Se, and Cu, respectively (Multimin 90, Axiota Animal Health, Fort Collins, CO, USA), and both were administered subcutaneously (1 mL/45 kg of BW) on the right side of the neck of each calf. Plasma samples were collected from a jugular vein on days 0, 3, 8, 15, 51, and 100. *Shows differences (*P* ≤ 0.05) between treatments. The means reported were adjusted for a covariate (baseline data from day 0).

**Figure 2.**
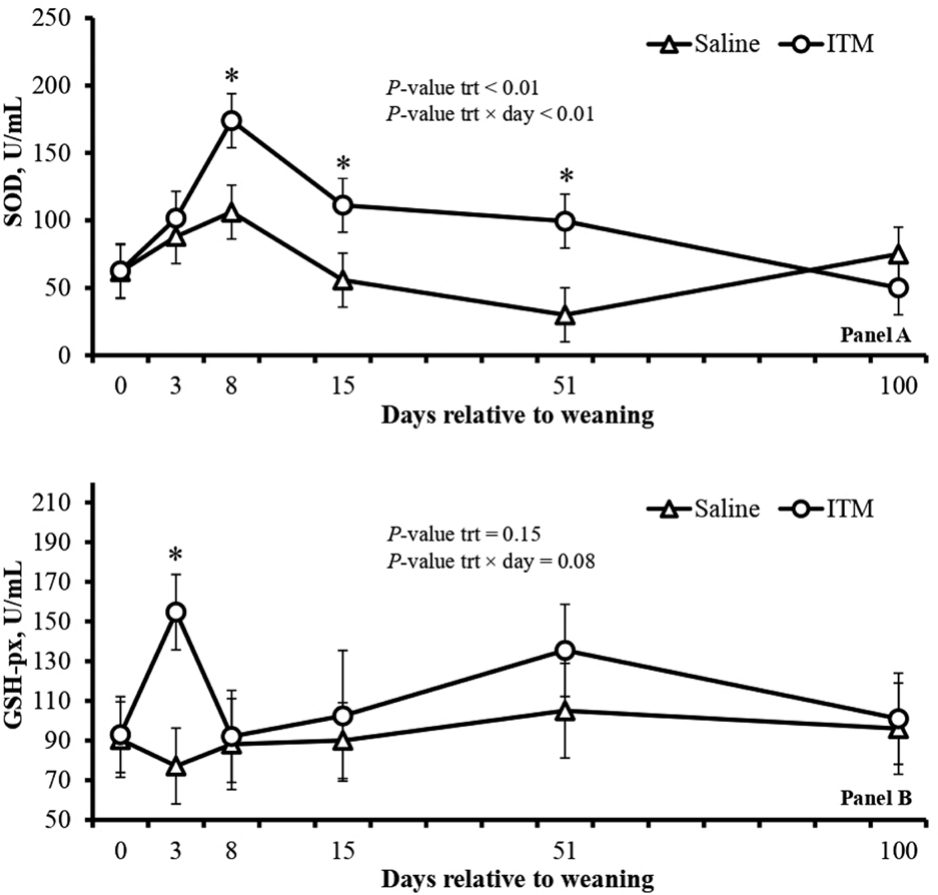
Plasma superoxide dismutase (SOD; A) and glutathione peroxidase (GSH-px; B) of Nellore calves receiving saline solution (saline; *n* = 14) or ITM (*n* = 14) at weaning (day 0) in experiment 1. The saline solution consisted of 0.9% NaCl, whereas ITM had 60, 10, 5, and 15 mg/mL of Zn, Mn, Se, and Cu, respectively (Multimin 90, Axiota Animal Health, Fort Collins, CO, USA), and both were administered subcutaneously (1 mL/45 kg of BW) on the right side of the neck of each calf. Plasma samples were collected from a jugular vein on days 0, 3, 8, 15, 51, and 100. *Shows differences (*P* ≤ 0.05) between treatments. The means reported were adjusted for a covariate (baseline data from day 0).

No effects of treatment or treatment × day were detected (*P *≥ 0.56) for serum concentration of Zn and Cu (Table 3). The ITM calves had lower (treatment × day effect; *P* = 0.02) serum cortisol concentrations on days 3 and 8 (Figure 1A) and lower (treatment × day effect; *P* = 0.01), plasma haptoglobin concentration on day 3 (Figure 1B) compared to Saline calves. No effects of treatment or treatment × day were detected (*P *≥ 0.69) for plasma concentration of ceruloplasmin (Figure 1C) in experiment 1. In addition, ITM calves had greater (treatment × day effect; *P* ≤ 0.01) plasma concentrations of SOD on days 8, 15 and 52 (Figure 2A), and tended to have greater (treatment × day effect; *P* = 0.08) plasma GSH-px concentration on day 3 (Figure 2B) compared to Saline calves.

**Table 3.** TM status of beef calves receiving saline solution (saline) or ITM at weaning (day 0) on experiments 1 and 2^1^

Items	Treatments^2^		SEM	*P*-value	
	Saline	ITM		Treatment	Treatment × day
Exp. 1, *n*	14	14			
Serum mineral concentration^3^					
Zn, μg/dL	66.8	73.8	7.52	0.64	0.56
Cu, μg/dL	57.3	57.6	2.70	0.92	0.87
Exp. 2, n	12	12			
Liver mineral concentration^4^					
Zn, μg/g	157	161	11.4	0.82	0.48
Cu, μg/g			9.89	<0.01	<0.01
Day 0	155	155			
Day 14	179^b^	269+			
Day 197	144	158			
Se, μg/g			0.63	0.08	0.03
Day 0	8.09	8.14			
Day 14	6.92^b^	10.1^a^			
Day 197	1.21	1.23			
Mn, μg/g	8.99	9.58	0.27	0.17	0.55
Fe, μg/g	351	345	26.6	0.76	0.57

^1^Experiment 1 was conducted at the farm school of the UFMS, Terenos, MS, Brazil, and used 86 weaned Nellore calves (40 females and 46 males). Experiment 2 was conducted at Dean Lee Research and Extension Center (Alexandria, LA, USA) and Iberia Research Station (Jeanerette, LA, USA) from Louisiana State University AgCenter and used 50 weaned male Brangus calves.

^2^Saline solution consisted of 0.9% NaCl, whereas ITM had 60, 10, 5, and 15 mg/mL of Zn, Mn, Se, and Cu, respectively (Multimin 90, Axiota Animal Heath, Fort Collins, CO, USA), and both were administered subcutaneously (1 mL/45 kg of BW) on the right side of the neck of each calf.

^3^Serum samples were collected from a jugular vein on days 0, 15, and 51.

^4^Liver samples were collected between the 10th and 11th intercostal space using a Tru-Cut biopsy needle on days 0, 14, and 197.

^a-b^Within a row, without a common superscript differ (*P* ≤ 0.05) or tends to differ (*P* ≤ 0.10). The means reported were adjusted for a covariate (baseline data from day 0).

The ITM calves showed reduced (treatment × day effect; *P* = 0.01) seeking time on days 1 and 4 (Table 4), compared to the Saline calves. No effects of treatment or treatment × day were detected (*P *≥ 0.14) for the other pasture behavior variables evaluated (Table 4). The ITM application tended to increase (treatment × day effect; *P* = 0.10) the IBR titers concentration on days 15 and 51 (Table 5). No effects of treatment or treatment × day were detected (*P* ≥ 0.50) for the titer concentration of PI_3_, BVDV-1, and BVDV-2. Furthermore, no effects of treatment or treatment × day were detected (*P *≥ 0.12) for BW or average daily gain (ADG) in experiment 1 (Table 6).

**Table 4.** Pasture behavior of beef calves receiving saline solution (Saline) or ITM at weaning (day 0) on experiment 1^1^

Items, % of the activities^3^	Treatments^2^		SEM	*P*-value	
	Saline	ITM		Treatment	Treatment × day
Exp. 1, *n*	14	14			
Grazing	37.7	37.4	1.29	0.87	0.42
Eating concentrate	2.49	2.46	0.32	0.94	0.82
Drinking water	1.02	0.87	0.18	0.58	0.14
Walking	9.55	8.88	0.87	0.59	0.72
Lying	21.5	22.0	1.14	0.79	0.29
Lying ruminating	3.46	3.26	0.42	0.73	0.82
Total lying	25.0	25.3	1.31	0.90	0.20
Standing	20.8	22.4	1.07	0.29	0.24
Standing ruminating	2.85	2.23	0.31	0.16	0.68
Total standing	23.6	24.6	1.14	0.54	0.29
Total ruminating	6.32	5.50	0.48	0.24	0.89
Playing	0.21	0.22	0.07	0.99	0.35
Vocalizing	0.14	0.24	0.07	0.43	0.69
Seeking			0.26	0.04	0.01
Day 1	3.37^a^	1.71^b^			
Day 2	0.41	0.41			
Day 4	0.10^a^	0.01^b^			
Day 5	0.01	0.01			
Day 6	0.21	0.20			
Day 7	0.01	0.01			
Day 9	0.01	0.01			

^1^Experiment 1 was conducted at the farm school of the UFMS, Terenos, MS, Brazil, and used 86 weaned Nellore calves (40 females and 46 males).

^2^Saline solution consisted of 0.9% NaCl, whereas ITM had 60, 10, 5, and 15 mg/mL of Zn, Mn, Se, and Cu, respectively (Multimin 90, Axiota Animal Heath, Fort Collins, CO, USA), and both were administered subcutaneously (1 mL/45 kg of BW) on the right side of the neck of each calf.

^3^On day 0, calves were individually identified on both sides of the body, with large numbers, using hair dye. An observation tower (elevated 5 m from the ground) was used to facilitate the visualization of the animals by the evaluators. The diurnal behavior of the animals was evaluated from 0630 h to 1800 h; with a 10-min interval between each scan on days 1, 2, 4, 5, 6, 7, and 9. Total time evaluated = 678 min/day.

^a-b^Within a row, without a common superscript differ (*P* ≤ 0.05) or tends to differ (*P* ≤ 0.10).

**Table 5.** Response to vaccination of beef calves receiving saline solution (saline) or ITM at weaning (day 0) on experiments 1 and 2^1^

Items^2^	Treatments^3^		SEM	*P*-value	
	Saline	ITM		Treatment	Treatment × day
Exp. 1, *n*	14	14			
IBR titers, log_2_			0.47	0.05	0.10
Day 0	0.00	0.00			
Day 15	3.83^b^	5.33^a^			
Day 51	3.67^b^	5.17^a^			
PI_3_ titers, log_2_	3.20	3.36	0.61	0.78	0.50
BVDV-1 titers, log_2_	1.83	2.22	0.60	0.66	0.62
BVDV-2 titers, log_2_	1.17	1.17	0.42	1.00	1.00
Exp. 2, *n*	12	12			
IBR titers, log_2_	3.74	3.90	0.19	0.58	0.31
PI_3_ titers, log_2_	7.23	7.60	0.18	0.20	0.38
BVDV-1, titers, log_2_	5.73	5.35	0.30	0.40	0.34
BVDV-2 titers, log_2_	6.76	7.29	0.22	0.05	0.72
BRSV titers, log_2_	3.04	3.95	0.47	0.23	0.97

^1^Experiment 1 was conducted at the farm school of the UFMS, Terenos, MS, Brazil, and used 86 weaned Nellore calves (40 females and 46 males). Experiment 2 was conducted at Dean Lee Research and Extension Center (Alexandria, LA, USA) and Iberia Research Station (Jeanerette, LA, USA) from Louisiana State University AgCenter and used 50 weaned male Brangus calves.

^2^On day 0, before treatment application, calves were vaccinated against IBR, parainfluenza-3 (PI_3_) virus, bovine viral diarrhea virus types 1 (BVDV-1) and 2 (BVDV-1), BRSV, and *Mannheimia haemolytica* (2 mL s.c.; Bovi Shield Gold One Shot, Zoetis). Serum samples were collected from a jugular vein on days 0, 15, and 51 in Experiment 1 and on days 0, 14, and 44 on Experiment 2.

^3^Saline solution consisted of 0.9% NaCl, whereas ITM had 60, 10, 5, and 15 mg/mL of Zn, Mn, Se, and Cu, respectively (Multimin 90, Axiota Animal Heath, Fort Collins, CO, USA), and both were administered subcutaneously (1 mL/45 kg of BW) on the right side of the neck of each calf.

^a-b^Within a row, without a common superscript differ (*P* ≤ 0.05) or tends to differ (*P* ≤ 0.10). The means reported were adjusted for a covariate (baseline data from day 0).

**Table 6.** Growth performance of beef calves receiving saline solution (saline) or ITM at weaning (day 0) on experiments 1 and 2^1^

Items	Treatments^2^		SEM	*P*-value	
	Saline	ITM		Treatment	Treatment × day
Exp. 1, *n*	43	43			
Body weight, kg			0.94	0.78	0.31
Day 0	198	198			
Day 8	197	195			
Day 15	196	195			
Day 51	202	203			
Day 100	195	196			
Average daily gain, kg/d			0.08	0.61	0.12
Days 0 to 8	–0.095	–0.339			
Days 8 to 15	–0.112	–0.011			
Days 15 to 51	0.169	0.217			
Days 51 to 100	–0.144	–0.140			
Average	–0.045	–0.069			
Exp. 2, *n*	25	25			
Body weight, kg			3.29	0.93	0.43
Day 0	264	264			
Day 14	263	265			
Day 44	260	265			
Day 78	264	268			
Day 122	253	253			
Day 162	268	260			
Day 197	293	291			
Average daily gain, kg/d			0.09	0.57	0.29
Days 0 to 14	–0.08	0.07			
Days 14 to 44	–0.10	0.00			
Days 44 to 78	0.11	0.07			
Days 78 to 122	–0.28	–0.33			
Days 122 to 162	0.36	0.17			
Days 162 to 197	0.73	0.88			
Average	0.12	0.15			

^1^Experiment 1 was conducted at the farm school of the UFMS, Terenos, MS, Brazil, and used 86 weaned Nellore calves (40 females and 46 males). Experiment 2 was conducted at Dean Lee Research and Extension Center (Alexandria, LA, USA) and Iberia Research Station (Jeanerette, LA, USA) from Louisiana State University AgCenter and used 50 weaned male Brangus calves.

^2^Saline solution consisted of 0.9% NaCl, whereas ITM had 60, 10, 5, and 15 mg/mL of Zn, Mn, Se, and Cu, respectively (Multimin 90, Axiota Animal Heath, Fort Collins, CO, USA), and both were administered subcutaneously (1 mL/45 kg of BW) on the right side of the neck of each calf.

^a-b^Within a row, without a common superscript differ (*P* ≤ 0.05) or tends to differ (*P* ≤ 0.10).

### Experiment 2.

The ITM calves had greater (treatment × day effect; *P* ≤ 0.03) liver concentrations of Cu and Se on day 14 (Table 3), compared to Saline calves. No effects of treatment or treatment × day were detected (*P *≥ 0.17) for liver concentrations of Zn, Mn, and Fe (Table 3).

The ITM application increased (treatment effect; *P* = 0.05) the BVDV-2 titers concentration, compared to the Saline application (Table 5). No effects of treatment or treatment × day were detected (*P* ≥ 0.20) for the titer concentration of IBR, PI_3_, BVDV-1, and BRSV (Table 5). Furthermore, no effects of treatment or treatment × day were detected (*P *≥ 0.29) for BW or ADG (Table 6).

## DISCUSSION

The ITM is a convenient method for rapidly enhancing TM status (Pogge et al., 2012), antioxidant capacity, and the immune system in beef calves post-weaning (Arthington and Havenga, 2012; Vedovatto et al., 2020). However, in experiment 1, ITM administration did not increase serum concentrations of Cu and Zn. This outcome may be attributed to the timing of sample collection relative to the peak TM concentrations in the bloodstream. Pogge et al. (2012) observed that TM concentrations peak approximately 10 to 12 hours post-injection and return to baseline levels within 24 hours. In our study, samples were collected on day 3 post-injection, potentially missing the peak concentration period. In addition, blood is not a reliable tissue to monitor TM status, as is the liver (Pogge et al., 2012).

In experiment 2, ITM successfully increased the liver Cu and Se status for at least 14 d after the injection. Several other experiments have reported an increase in liver TM concentration after an ITM application, variating the specifics minerals increased and also the storage time (Pogge et al., 2012; Arthington et al., 2014, Genther-Schroeder and Hansen, 2014, 2015; Hernandez et al., 2024). The differences between experiments are probably related to the status of each TM at the beginning of the experiment and the TM concentration in the diet consumed during the period evaluated.

Calves that received ITM demonstrated alleviated stress-related responses post-weaning, as evidenced by decreased plasma concentration of cortisol and haptoglobin, as well as reduced seeking time compared to the saline treatment. These variables show that ITM vs. saline calves were calmer and suffered less stress by weaning. Differences in behavior were also observed by Rauch et al. (2019), but conversely, to our study, the ITM vs. saline application increased the time walking after weaning (indicating an exploratory behavior) in abruptly weaned calves. Although the specific pathways or mechanisms through which ITM influences behavior and stress are currently unknown, in an observational study with humans, high cortisol levels were associated with oxidative stress (Kim et al., 2022). A high concentration of reactive oxygen species affects the function of the adrenal cortex (Prasad et al., 2014) and, consequently, cortisol production. In experiment 1, ITM application increased the plasma concentration of the antioxidant enzymes SOD and GSH-px and potentially decreased the oxidative stress on those animals. This improvement in the antioxidant system by ITM application was likely responsible for reducing cortisol production and, consequently, the haptoglobin concentration and affecting behavior.

An increase in SOD and GSH-px concentration by ITM application was also observed in three other experiments conducted by our group (Vedovatto et al., 2019a, 2019b, 2020). The minerals Zn, Mn, and Cu are components of the SOD enzymes found in the form of Cu/Zn-SOD, and Mn-SOD and are responsible for catalyzing the dismutation of the superoxide anion to molecular oxygen and hydrogen peroxide (Markclund, 1980). In addition, Se is a component of the GSH-px enzyme whose main function is to catalyze the reduction of hydroperoxides, such as hydrogen peroxides and lipid hydroperoxides (Rotruck et al., 1973). Thus, as the TM in the formula applied are components of these enzymes, an increase in its concentration is expected in animals receiving an ITM application.

In experiment 1, ITM did not affect the plasma concentration of ceruloplasmin but decreased the plasma concentration of haptoglobin. Conversely, Arthington et al. (2014) observed an increase in haptoglobin and ceruloplasmin in calves receiving ITM vs. saline injections following weaning and transport. Further, several other experiments observed no differences in haptoglobin or ceruloplasmin between animals receiving ITM vs. saline injections (Caramalac et al., 2021; Hernandez et al., 2024; Vedovatto et al., 2019a, 2019b). As mentioned previously, differences between studies are probably related to the magnitude of stress, management, and TM status. The decrease in the concentration of haptoglobin in ITM calves in experiment 1, could be a consequence of less cortisol production, as cortisol induces inflammatory responses (Gouvêa et al., 2022).

Nevertheless, the positive effects of ITM on the health and immune system of cattle are more prevalent among the studies (Teixeira et al., 2014; Vedovatto et al., 2020). In challenging scenarios, such as weaning and transportation, TM supplementation may become particularly important, as these highly stressed cattle are more likely to develop diseases after the challenge. Therefore, the impact of TM on immune function is beneficial for producing an effective immune response to pathogens.

In the current experiments, the TMI affected the humoral immune response. The ITM vs. saline calves had greater serum concentrations of neutralizing antibody titers against IBR (experiment 1) and BVDV-2 (experiment 2). Serum titers are used to indicate antibody protection against a specific pathogen, thereby indicating vaccine efficacy (Rosenbaum et al., 1970). A practical biological value associated with a rapid onset of the immune response as increasing antibody titers is related to the effectivity of the immune system in limiting disease progression. The IBR and BVDV are agents that may relate to bovine respiratory disease (Martin and Bohac, 1986), which is the most economically important disease in newly received beef cattle (Edwards, 2010), and the leading cause of morbidity and mortality in feedlots (Woolums et al., 2005).

The mechanisms by which ITM improved the immune system in our experiments are probably related to the effects of reduced cortisol and increased antioxidant enzyme concentration. Cortisol is known to be a suppressor of the immune system by reducing B-lymphocyte cell antibody production and neutrophil migration, which consequently impacts the production of antibodies (Kadmiel and Cidlowski, 2013). In addition, the increased production of SOD and GSH-px following ITM is possibly reflected in lower lipid peroxidation and cellular damage to leukocytes, affecting the immune response (Vedovatto et al., 2020). Furthermore, Zn is essential for highly proliferating cells, especially in the immune system, and Cu stimulates neutrophil and monocyte responses (Maggini et al., 2007). In addition, Se is a component of the enzyme thioredoxin reductase, that affects the redox regulation of several key enzymes, transcription factors, and receptors, that affect the expression of genes encoding proteins involved in immune response (Maggini et al., 2007). Thus, the combination of less cortisol and greater antioxidant concentrations led to an increased response to vaccination in ITM calves.

The ITM application did not affect the growth in our experiments. This lack of differences in performance could be attributed to the adequate mineral status of the calves during the study, and thus, it was likely not a limiting factor for growth. Literature data on the effects of supplementation of ITM on growth performance are quite variable. Inconsistencies of ITM responses are attributed to many factors, including the magnitude of stress, breed, and mainly nutritional and TM status. One experiment has reported greater growth performance in calves supplemented with ITM at weaning (Rauch et al., 2019), while others did not detect differences (Vedovatto et al., 2020) or even reduced growth performance (Arthington et al., 2014; Zornitta et al., 2022) compared to control groups. In an experiment with yearling Angus crossbred steers in a feedlot, only animals TM deficient had improved BW after an ITM application, but not animals with an adequate TM status (Genther and Hansen, 2014) which corroborates our explanation for no improvement in BW by ITM application in our experiment.

In the current experiments, the greatest deficiencies were protein and energy, and this explains the lack of growth during the entire experimental period in experiment 1 and for more than 4 mo in experiment 2. Thus, despite the higher supply of TM through ITM, the high deficiency of protein and energy may have limited growth and prevented ITM-induced increase in calf growth performance, as observed by Vedovatto et al. (2020) and Zornitta et al. (2022).

## CONCLUSION

The ITM application for *B. indicus or B. indicus*-influenced calves at weaning improved Cu and Se status, enhanced antioxidant and immune responses, and reduced stress and inflammation, though it did not affect growth.

## References

[CIT0001] AOAC. 2006. Official methods of analysis. 18th ed. Gaithersburg (MD): Association of Official Analytical Chemists.

[CIT0002] Arthington, J. D., and L. J. Havenga. 2012. Effect of injectable trace minerals on the humoral immune response to multivalent vaccine administration in beef calves. J. Anim. Sci. 90:1966–1971. doi: https://doi.org/10.2527/jas.2011-4024 22178855

[CIT0003] Arthington, J. D., P. Moriel, P. G. M. A. Martins, G. C. Lamb, and L. J. Havenga. 2014. Effects of trace mineral injections on measures of performance and trace mineral status of pre- and postweaned beef calves. J. Anim. Sci. 92:2630–2640. doi: https://doi.org/10.2527/jas.2013-7164 24867937

[CIT0004] Arthington, J. D., and J. Ranches. 2021. Trace mineral nutrition of grazing beef cattle. Animals. 11:2767. doi: https://doi.org/10.3390/ani11102767 34679788 PMC8532955

[CIT0005] Caramalac, L. S., P. Moriel, J. Ranches, G. M. Silva, and J. D. Arthington. 2021. Comparison of injectable trace minerals vs. adjuvant on measures of innate and humoral immune responses of beef heifers. Livestock Sci. 251:104665. doi: https://doi.org/10.1016/j.livsci.2021.104665

[CIT0006] Cooke, R. F., and J. D. Arthington. 2013. Concentrations of haptoglobin in bovine plasma determined by ELISA or a colorimetric method based on peroxidase activity: methods to determine haptoglobin in bovine plasma. J. Anim. Physiol. Anim. Nutr. (Berl). 97:531–536. doi: https://doi.org/10.1111/j.1439-0396.2012.01298.x 22487219

[CIT0007] Demetriou, J. A., P. A. Drewes, and J. B. Gin. 1974. Ceruloplasmin. Pages 857–846 in clinical chemistry: principles and techniques. 2nd ed. Hagerstown (MD): Harper and Row.

[CIT0008] Edwards, T. A. 2010. Control methods for bovine respiratory disease for feedlot cattle. Vet. Clin. North Am. Food Anim. Pract. 26:273–284. doi: https://doi.org/10.1016/j.cvfa.2010.03.005 20619184

[CIT0009] Enríquez, D. H., R. Ungerfeld, G. Quintans, A. L. Guidoni, and M. J. Hötzel. 2010. The effects of alternative weaning methods on behaviour in beef calves. Livestock Sci. 128:20–27. doi: https://doi.org/10.1016/j.livsci.2009.10.007

[CIT0010] Genther, O. N., and S. L. Hansen. 2014. A multielement trace mineral injection improves liver copper and selenium concentrations and manganese superoxide dismutase activity in beef steers. J. Anim. Sci. 92:695–704. doi: https://doi.org/10.2527/jas.2013-7066 24398829

[CIT0011] Genther-Schroeder, O. N., and S. L. Hansen. 2015. Effect of a multielement trace mineral injection before transit stress on inflammatory response, growth performance, and carcass characteristics of beef steers. J. Anim. Sci. 93:1767–1779. doi: https://doi.org/10.2527/jas.2014-8709 26020198

[CIT0012] Gouvêa, V. N., R. F. Cooke, and R. S. Marques. 2022. Impacts of stress-induced inflammation on feed intake of beef cattle. Front. Anim. Sci. 3:962748. doi: https://doi.org/10.3389/fanim.2022.962748

[CIT0013] Hernandez, G. P., M. F. Ferreira, A. C. Santos, D. Bohnert, and J. Ranches. 2024. Effects of trace mineral injections on measures of growth and trace mineral status of primiparous cows and their calves. Transl. Anim. Sci. 8:txae068. doi: https://doi.org/10.1093/tas/txae068 38774510 PMC11107845

[CIT0014] Kadmiel, M., and J. A. Cidlowski. 2013. Glucocorticoid receptor signaling in health and disease. Trends Pharmacol. Sci. 34:518–530. doi: https://doi.org/10.1016/j.tips.2013.07.003 23953592 PMC3951203

[CIT0015] Kim, J., K. Yun, A. Cho, D. H. Kim, Y. K. Lee, M. J. Choi, S. Kim, H. Kim, J. W. Yoon, and H. C. Park. 2022. High cortisol levels are associated with oxidative stress and mortality in maintenance hemodialysis patients. BMC Nephrol. 23:98. doi: https://doi.org/10.1186/s12882-022-02722-w 35260104 PMC8903641

[CIT0016] Maggini, S., E. S. Wintergerst, S. Beveridge, and D. H. Hornig. 2007. Selected vitamins and trace elements support immune function by strengthening epithelial barriers and cellular and humoral immune responses. Br. J. Nutr. 98:S29–S35. doi: https://doi.org/10.1017/s0007114507832971 17922955

[CIT0017] Markclund, S. 1980. Distribution of CuZn superoxide dismutase and Mn superoxide dismutase in human tissues and extracelular fuids. Acta Physiol. Scand. 492:19–23. PMID: 6939305. 6939305

[CIT0018] Marques, R. S., R. F. Cooke, C. L. Francisco, and D. W. Bohnert. 2012. Effects of twenty-four hour transport or twenty-four hour feed and water deprivation on physiologic and performance responses of feeder cattle. J. Anim. Sci. 90:5040–5046. doi: https://doi.org/10.2527/jas.2012-5425 22851237

[CIT0019] Martin, S. W., and J. G. Bohac. 1986. The association between serological titers in infectious bovine rhinotracheitis virus, bovine virus diarrhea virus, parainfluenza-3 virus, respiratory syncytial virus and treatment for respiratory disease in Ontario feedlot calves. Can. J. Vet. Res. 50:351–358. PMID: 3017529. 3017529 PMC1255225

[CIT0020] Mullis, L. A., J. W. Spears, and R. L. McCraw. 2003. Effects of breed (Angus vs Simmental) and copper and zinc source on mineral status of steers fed high dietary iron. J. Anim. Sci. 81:318–322. doi: https://doi.org/10.2527/2003.811318x 12597403

[CIT0021] NASEM. 2016. Nutrient requirements of beef cattle: 8th rev. ed. Washington (DC): National Acad. Press.

[CIT0022] Pogge, D. J., E. L. Richter, M. E. Drewnoski, and S. L. Hansen. 2012. Mineral concentrations of plasma and liver after injection with a trace mineral complex differ among Angus and Simmental cattle. J. Anim. Sci. 90:2692–2698. doi: https://doi.org/10.2527/jas.2012-4482 22896735

[CIT0023] Prasad, R., J. C. Kowalczyk, E. Meimaridou, H. L. Storr, and L. A. Metherell. 2014. Oxidative stress and adrenocortical insufficiency. J. Endocrinol. 221:R63–R73. doi: https://doi.org/10.1530/joe-13-0346 24623797 PMC4045218

[CIT0024] Ranches, J., R. Alves, M. Vedovatto, E. A. Palmer, P. Moriel, and J. D. Arthington. 2021. Differences in copper and selenium metabolism between Angus (*Bos taurus*) and Brahman (*Bos indicus*) cattle. J. Anim. Sci. 99:skab048. doi: https://doi.org/10.1093/jas/skab048 33585942 PMC7947969

[CIT0025] Rauch, J. C., R. S. Stokes, and D. W. Shike. 2019. Evaluation of two-stage weaning and trace mineral injection on receiving cattle growth performance and behavior. Transl Anim Sci 3:155–163. doi: https://doi.org/10.1093/tas/txy131 32289111 PMC7107296

[CIT0026] Richeson, J. T., and E. B. Kegley. 2011. Effect of supplemental trace minerals from injection on health and performance of highly stressed, newly received beef heifers. Prof. Anim. Sci. 27:461–466. doi: https://doi.org/10.15232/s1080-7446(15)30519-2

[CIT0027] Rosenbaum, M. J., E. A. Edwards, and E. J. Sullivan. 1970. Micromethods for respiratory virus sero-epidemiology. Health Lab Sci. 7:42–52. PMID: 4313274. 4313274

[CIT0028] Rotruck, J. T., A. L. Pope, H. E. Ganther, A. B. Swanson, D. G. Hafeman, and W. G. Hoekstra. 1973. Selenium: biochemical role as a component of glutathione peroxidase. Science 179:588–590. doi: https://doi.org/10.1126/science.179.4073.588 4686466

[CIT0029] Sordillo, L. M., and S. L. Aitken. 2009. Impact of oxidative stress on the health and immune function of dairy cattle. Vet. Immunol. Immunopathol. 128:104–109. doi: https://doi.org/10.1016/j.vetimm.2008.10.305 19027173

[CIT0030] Spears, J. W., and E. B. Kegley. 2002. Effect of zinc source (zinc oxide vs. zinc proteinate) and level on performance, carcass characteristics, and immune response of growing and finishing steers. J. Anim. Sci. 80:2747–2752. doi: https://doi.org/10.2527/2002.80102747x 12413098

[CIT0031] Teixeira, A. G. V., F. S. Lima, M. L. S. Bicalho, A. Kussler, S. F. Lima, M. J. Felippe, and R. C. Bicalho. 2014. Effect of an injectable trace mineral supplement containing selenium, copper, zinc, and manganese on immunity, health, and growth of dairy calves. J. Dairy Sci. 97:4216–4226. doi: https://doi.org/10.3168/jds.2013-7625 24835970

[CIT0032] Van Soest, P. J., J. B. Robertson, and B. A. Lewis. 1991. Methods for dietary fiber, neutral detergent fiber, and nonstarch polysaccharides in relation to nutrition animal. J. Dairy Sci. 74:3583–3597. doi: https://doi.org/10.3168/jds.S0022-0302(91)78551-2 1660498

[CIT0033] Vedovatto, M., C. Da Silva Pereira, I. M. Cortada Neto, P. Moriel, M. D. G. Morais, and G. L. Franco. 2020. Effect of a trace mineral injection at weaning on growth, antioxidant enzymes activity, and immune system in Nellore calves. Trop. Anim. Health Prod. 52:881–886. doi: https://doi.org/10.1007/s11250-019-02056-0 31471881

[CIT0034] Vedovatto, M., P. Moriel, R. F. Cooke, D. S. Costa, F. J. C. Faria, I. M. Cortada Neto, A. L. L. Bento, R. F. A. T. Rocha, L. C. L. Ferreira, R. G. Almeida, et al 2019a. Effects of a single trace mineral injection at the beginning of the fixed-time AI treatment regimen on reproductive function and antioxidant response of grazing Nellore cows. Anim. Reprod. Sci. 211:106234. doi: https://doi.org/10.1016/j.anireprosci.2019.106234 31785632

[CIT0035] Vedovatto, M., P. Moriel, R. F. Cooke, D. S. Costa, F. J. C. Faria, I. M. Cortada Neto, C. S. Pereira, A. L. L. Bento, R. G. Almeida, S. A. Santos, et al 2019b. Effects of a single trace mineral injection on body parameters, ovarian structures, pregnancy rate, and components of the innate immune system of grazing Nellore cows synchronized to a fixed-time AI protocol. Livest. Sci. 225:123–128. doi: https://doi.org/10.1016/j.livsci.2019.05.011

[CIT0036] Vieira, D. G., M. Vedovatto, H. J. Fernandes, E. D. A. Lima, M. C. D’Oliveira, U. D. A. Curcio, J. Ranches, M. F. Ferreira, O. A. D. Sousa, B. I. Cappellozza, et al 2023. Effects of an appeasing substance application at weaning on growth, stress, behavior, and response to vaccination of Bos indicus calves. Animals. 13:3033. doi: https://doi.org/10.3390/ani13193033 37835638 PMC10571994

[CIT0037] Weiss, W. P., H. R. Conrad, and N. R. Pierre. 1992. A theoretically-based model for predicting total digestible nutrient values of forages and concentrates. J. Anim. Sci. 70:95–110. doi: https://doi.org/10.1016/0377-8401(92)90034-4

[CIT0038] Woolums, A. R., G. H. Loneragan, L. L. Hawkins, and S. M. Williams. 2005. Baseline management practices and animal health data reported by U.S. feedlots responding to a survey regarding acute interstitial pneumonia. Bovine Pract. 39:116–124. doi: https://doi.org/10.21423/bovine-vol39no2p116-124

[CIT0039] Zornitta, C., M. C. D’Oliveira, A. L. De Lucca Bento, R. F. A. T. Rocha, M. Vedovatto, and G. L. Franco. 2022. Effect of injectable trace mineral at weaning on growth and physiology of Nellore calves under feed restriction. Trop. Anim. Health Prod. 54:18. doi: https://doi.org/10.1007/s11250-021-03001-w 34910259

